# Supplementary material for: Are carbon emissions trading and green financial instruments synergistic? -Comprehensive quantitative research based on content analysis
Source: PLoS One. 2024 Mar 7;19(3):e0298601. doi: 10.1371/journal.pone.0298601 (PMC10919601; doi:10.1371/journal.pone.0298601)
Supplement: S1 Data — (ZIP) [file pone.0298601.s001.zip › Data/地级市Z_F工作报告.zip!/╣π╢1⁄2/╒┐╜¡╩╨/└┤╫╘╣1⁄2╓┌║┼í╛┬φ┐╦╩2╛▌═°í┐_2018.docx]

各位代表：

　　现在，我代表市人民政府向大会报告政府工作，请予审议，并请市政协各位委员和其他列席同志提出意见。

2017年工作回顾

　　2017年是本届政府履职施政的开局之年。一年来，在市委坚强领导下，在市人大、市政协监督支持下，我们坚持以习近平新时代中国特色社会主义思想为指导，深入贯彻党的十八大、十八届历次全会精神，认真学习贯彻党的十九大精神，认真贯彻落实习近平总书记关于广东工作重要批示和省委十二届二次、三次全会精神，认真落实市委十一届三次全会部署要求，扎实推进供给侧结构性改革，全力保障和改善民生，较好完成市十四届人大一次会议确定的目标任务。

　　全市经济运行稳中有进、持续向好。预计实现生产总值2850亿元，增长7.7%。规上工业增加值833亿元，增长9%。固定资产投资1642亿元，增长7.2%。社会消费品零售总额1579亿元，增长10.2%。外贸进出口总额340亿元，增长10%。实际利用外资8095万美元，增长32%。一般公共预算收入135亿元、增长21%；来源于湛江财政总收入583.8亿元、增长25.2%。湛江居民人均可支配收入19596元，增长9.3%。城镇登记失业率控制在3.5%以内。

　　2017年是国家和省持续重视湛江、赋予湛江崭新发展使命的机遇之年。国务院批复《北部湾城市群发展规划》，湛江被列为城市群“一核两极”之一的中心城市；批准《湛江城市总体规划》，湛江建设经济繁荣、和谐宜居、生态良好、富有活力、特色鲜明的现代化城市有了明确的规划指引；发布《全国“十三五”现代综合交通运输体系规划》，湛江从发展边陲、交通末梢，跃升全国性综合交通枢纽。省政府发布《广东省沿海经济带综合发展规划》，湛江被定位为省域副中心城市。省委十二届三次全会明确提出，把东西两翼沿海地区打造成广东新的增长极。湛江在国家和全省发展大局中的定位更加明晰、地位更加重要。

　　2017年是全市人民撸起袖子、拼搏进取的实干之年。在市委领导下，全市上下主动对标新定位、新要求，全力落实“三大抓手”实施方案和“五大产业发展计划”，着力优化经济结构，建成一批重大项目，办成一批重大实事，有效提升发展质量和效益。湛江国际机场飞行等级由4C升为4E，飞行跑道由2800米延长到3200米，由支线机场上升为广东第三大干线机场，引入国内外一流团队完成机场航站楼设计方案优化升级，一个现代化国际新机场呼之欲出；湛江高铁西客站主体框架工程全部完成，湛江人民期待已久的高铁开通在即；东海岛铁路湛江西至钢铁基地段竣工；汕湛高速湛江段建成通车，湛江与珠三角共建产业、共谋发展的合作之路更加顺畅、更加快捷；湛江港重振雄风，货物吞吐量突破2.8亿吨，回到全国沿海十大港口之列。湛江钢铁一期全面达产稳产，树起国内发展钢铁先进产能、深化供给侧结构性改革的标杆。中海油湛江分公司“南海高温高压钻完井关键技术及工业化应用”荣获国家科技进步一等奖，实现了我市国家级科技大奖“零”的突破，成为我市科技创新的典范。湛江中心人民医院结束79年老院区运作，整体迁至现代化大型新院区，占地面积从32亩增至216亩，床位从1300张增至2000张，较好实现了广大百姓对医疗环境提质优服的美好期待。2017中国海博会成功举办，展会规模更大，专业化、国际化水平更高，展示了湛江走向世界、开放发展的豪情与自信。隆重召开商会会长暨民营企业家座谈会，成功举办投资项目推介暨重大项目集中签约活动，大批乡贤踊跃回乡创业，一批央企、珠三角先进企业和世界500强企业看好湛江得天独厚的发展优势，看好湛江充满活力的发展前景，投资湛江信心增强、热情高涨，湛江振兴发展的机遇更多、态势更好、后劲更足！

　　一年来，我们主要做了以下工作。

　　（一）注重高水平谋划，交通建设实现新突破。坚持一手抓规划设计，一手抓项目建设，全力推动综合交通对外快速畅达、对内便捷成网。交通规划编制取得突破性进展。全力争取上级和有关方面最大程度支持，编制完成全市综合交通运输“十三五”发展规划，围绕“2小时通达珠三角、1小时贯通湛江全域”，明确加快实施“55811”综合交通工程；坚持海陆空铁“无缝衔接”，组织编制湛江铁路总图，推动高铁入城、高速入市，规划建设中心城区高铁枢纽站，推动空铁联运，机场、高铁“零换乘”；面向国际招投标，引进高水平设计团队，完成徐闻港南山作业区综合交通枢纽“海上升明月”、高铁西站和调顺跨海大桥建设方案优化设计，倾力打造代表广东形象、彰显湛江魅力的亮丽名片。交通重点项目建设取得突破性进展。完成基础设施建设投资497.5亿元、增长10.1%，其中交通建设投资132.2亿元、增长25.1%。湛江国际机场主体工程基础工作全面推进，空港经济区空铁一体化交通专项规划审议通过；湛江机场新增2条国际、4条国内航线，加密深湛往返航班，两地商旅往返更加便利。完成黎湛铁路电气化改造。高速公路通车里程新增67公里，累计突破300公里，玉湛高速、东雷高速全线动工，汕湛高速吴川支线开工建设；改造国省道57公里，大修县道125.9公里，改造乡道124公里，硬化村道684公里。湛江钢铁30万吨级配套码头建成投产，大唐雷州电厂配套码头主体工程基本完工，霞山港区通用码头开工建设；徐闻港“三大工程”顺利推进，琼州海峡北岸港航资源整合取得实质性进展。14个电网输变电工程建成运行。在重大交通基础设施项目带动下，全市184项重点项目完成投资392亿元，13个项目建成运行。

　　（二）注重提振实体经济，产业转型迈出新步伐。全面实施“五大产业发展计划”，注重盘活升级存量，全力引进优质增量，推动产业转型升级、协调发展。实体经济成本有效降低。出台43条措施支持企业降本增效，取消、停征35项涉企收费项目，降低企业综合成本63.8亿元。持续开展“暖企”行动，解决108个工业企业历史遗留问题。工业经济效益持续提升。完成工业投资627.3亿元，增长10.7%；新增规上工业企业100家。湛江钢铁1550冷轧生产线全线贯通，完成钢材产量688万吨，薄板热销国内主流汽车生产企业，厚板通过国内和出口欧美、日韩资质认证，多项经济效益和环保指标创下全国之最。中科炼化项目累计完成投资72.2亿元，主体工程完成全部基础设计审查，全面开展EPC招标。中海油湛江分公司完成开采1000万吨油气当量，东兴石化、晨鸣纸业等骨干企业经营效益良好。产业集聚发展态势良好。园区完成规上工业总产值1916亿元、增加值544亿元，分别增长31%和28%。12个省级产业园（集聚地）新落地项目51个，新投产25个。湛江产业转移工业园全省考核第一名。精准招商成果丰硕。先后与30多家国内和世界500强企业签署合作协议，投资近千亿元的69个合作项目吸引120多家中外企业和投资机构广泛关注，14个集中签约项目涉及投资和授信2350亿元。服务业升级发展成效明显。跨境电商公共服务平台上线运作，跨境电商产业园一期和中国移动广东（湛江）数据中心建成运营，电商企业超过3000家。引进中信银行等5家金融机构。港口集装箱吞吐量90万标箱，增长25%；民航旅客吞吐量209万人次，增长24.5%。旅游接待人数、旅游收入分别增长15%和20%。荣获全国旅游厕所革命先进市称号。

　　（三）注重遵循规律优化布局，城市扩容提质展现新面貌。中心城区推进实施26项市政工程，世贸人行天桥建成使用，海湾路等15条道路建成通车，疏港大道一期路面改造完成，西城快线加快建设。东菊公园建成。麻章日产7.5万立方米自来水厂投入运行。海东新区总体规划批准实施，完成开发投资175.7亿元，建成项目56个；调顺跨海大桥动工建设，体育路、金湾南路等16条道路建成通车。城市管理切实加强。完成“三旧”改造面积1637.9亩。出台城区市容和环境卫生管理等条例，国家卫生城市顺利通过复审，积极参加央视“魅力中国城”竞演，被联合国环境署授予“SUC项目可持续发展先锋城市”中国首批试点，城市知名度、美誉度进一步提升。

　　（四）注重夯实“三农”基础，现代农业与县域经济发展呈现新亮点。现代农业稳健发展。完成农业总产值838亿元，海洋经济总产值1446亿元。雷州半岛现代农业核心示范区入选首批广东农业公园。农作物良种覆盖率、水稻综合机械化率分别超过95%和70%。新增市级以上农业龙头企业26家、农业品牌56个。正大（湛江）公司“农场到餐桌”安全食品全产业链项目投产。完成水利建设投资18亿元，新增（恢复）灌溉面积18万亩。完成海堤加固达标45公里。农村土地承包经营确权登记颁证率90.2%，超额完成省下达目标。5361条自然村完成农村人居生态环境综合整治任务。有效推动县域发展园区经济，推进县城扩容提质。县域生产总值达到1559亿元，超过全市GDP总量50%。廉江市生产总值突破500亿元，成为“中国小家电产业基地”，龙健电子等新兴产业项目建成投产。雷州市完成5.3公里市政道路改造，建成全国单体规模最大的生物质燃料项目。吴川市基本完成湛江国际机场征拆，华昱产业转移园评为全省建设管理优秀园区。遂溪县稳步推进县城“两河三岸”综合整治开发。徐闻县5个重点工业项目建成投产，成为广东风电清洁能源基地。5县（市）、37个乡镇污水处理设施建成使用。安铺镇、南三镇和麻章镇分别成为全国、全省特色小镇创建示范点。

　　（五）注重增强内生动力，有效释放发展新能量。启动实施179项改革事项，积极推进重点领域和关键环节改革。深入推进“放管服”改革。完成1177项行政审批标准化编制，更新52个政府部门权责清单6322项行政职权，取消调整行政审批620项，委托下放163项，清理规范行政审批中介服务事项35项。9家企业成为项目投资建设直接落地改革试点。“一门式、一网式”政府服务全面推广，1934个村（社区）公共服务平台建成运作。网上办事大厅受理审批事项办结率99%。城市信用指数排名全国地级市前十、全省第一。推行“多证合一”“证照分离”和工商登记便利化改革，发放电子营业执照4521份。新登记市场主体增长17.9%，新登记民营企业增长26.2%。民间投资1100亿元，增长10%；民营经济增加值1750亿元，增长8%。创新发展活力显现。积极培育发展创新主体，新增高新技术企业54家，增长70%。新增18家省级工程技术研究中心、2家省产业技术创新联盟、2家省级众创空间。获省科学技术奖12项，居粤东西北第一。国家知识产权试点城市通过验收，技术合同、交易额分别增长31%和84.7%。“湛江海洋生物创新型产业集群”成为全省试点，21个海洋经济创新发展示范市项目快速推进。成功举办第三届中欧生命科学论坛。对外开放步伐加快。2017中国海博会吸引全球63个国家、全国21个省（市、自治区）、46个沿海城市、3200多家企业参展参会，成功举办高端论坛7个。成功举办中国水博会、东盟·农博会。国际贸易“单一窗口”国家标准版率先落地湛江，宝满、东海岛港区扩大开放获得批准。推广关企联网、在线通关，进口货物通关时间压缩三分之一以上。积极落实《北部湾城市群发展规划》，推动北部湾城市实现住房公积金异地互认互提互贷、异地就医直接结算，落实旅游景点套票优惠，发行北部湾旅游护照；成功举办首届北部湾城市运动会，北部湾中心城市的影响力进一步提升。恒兴集团投资的埃及国家水产产业园建成投产，湛江水产企业成功输出“中国标准”。 新增英国朴茨茅斯、泰国攀牙府两个国际友好交流城市，我市国际友城及友好交流城市达到17个。

　　（六）注重补短板办实事，社会民生得到新改善。市财政社会民生支出349.3亿元，占一般公共预算支出77.6%。全力开展精准扶贫。落实扶贫资金43亿元，实施扶贫项目11.2万个，7.2万贫困人口实现脱贫。解决120万人通自来水和饮水安全问题。民生保障持续加强。十件民生实事基本完成，十大民心工程顺利推进。新增城镇就业7.9万人、失业人员再就业3.5万人。城乡居民基本养老保险参保率99%，新增养老床位6218张。企业职工养老保险金人均提高5.5%。实现跨省异地就医直接结算，门诊诊查费纳入医保报销范围。建成市残疾人康复中心。完成9400户农村危房、4269户棚户区改造，提高孤儿、残疾人、低保对象等困难群众救助标准。社会事业不断进步。扎实推进教育现代化，霞山成为“广东省推进建设教育现代化县（市、区）”；新建扩建9所市区学校、新增学位11700个。争取上级20亿元资金，实施518个医疗卫生升级项目。新增医疗床位1615张，升级改造7间中心卫生院，实现“县有中医院、镇有中医馆”，县域住院率超过80%。120数字化指挥中心投入使用。援藏医生团队荣获国家“最美医生团队”。雷剧《岭南圣母》等作品获广东省第十届精神文明建设“五个一工程”优秀文艺作品奖，遂溪醒狮亮相丝绸之路国际电影节。广东电网湛江供电局等2个单位成为全国文明单位，遂溪陈家村等3个村成为全国文明村，张志添等3人被评为“中国好人”。获得首届北部湾城市运动会团体总分第一，湛江聋人女足代表国家出征第23届夏季听障奥运会，湛江籍运动员朱倩获得听障奥运女子4×200自由泳接力赛银牌。粮食、物价、民族宗教、档案、对台、人防、残疾人、地方志、气象、地震等工作取得新成绩。社会大局和谐稳定。成功应对防御台风“卡努”正面袭击。深入开展“飓风2017”等专项行动，有效打击黄赌毒黑拐骗等违法犯罪。深入实施“平安细胞”工程，健全街面联防巡控“四项机制”，市区“两抢”警情下降43.6%。基层反走私综合治理经验在全国推广。有效化解信访积案185宗。扎实推进食品药品安全工程。从严落实安全生产主体责任，连续9年没有发生重特大安全生产事故。

　　（七）注重坚守绿色底线，生态文明建设实现新提升。坚定绿色发展不动摇，获得全省环境保护责任考核优秀等次。“湛江蓝”成为常态。持续防治大气污染，淘汰黄标车10547辆、燃煤锅炉30台，环境空气综合质量指数位居全省第二。治水成效明显。率先在全省试点“湾长制”，海湾清理整治初见成效。健全“河长制”，深化九洲江－鹤地水库、小东江流域综合整治，市域大江大河、湖库水质总体良好，城市饮用水源水质100%达标。环保执法力度加大。责令整改企业36家、立案处罚107家。完成5项中央环保督察整改任务，积极配合国家海洋督察整改。新一轮绿化大行动和雷州半岛生态修复持续推进。实施森林碳汇等林业“四大”重点工程，完成造林绿化7.7万亩，绿化美化乡村193条。雷州湾湿地成为“候鸟天堂”。

　　（八）注重实干转变作风，政府建设取得新成效。坚持以习近平新时代中国特色社会主义思想统领政府工作，认真学懂弄通做实党的十九大精神。政风进一步转变。政府系统“两学一做”学习教育常态化，深入开展“机关作风建设强化年”活动，全面落实中央八项规定精神，驰而不息纠正“四风”，全市“三公”经费和会议费支出下降9.8%。全面推行“两函两书”制度，政府督查督办有效加强。依法行政水平进一步提升。建立政府法律顾问、立法咨询暨重大行政决策咨询论证专家库，清理规范性文件5464件，获得省依法行政考评良好等次。自觉接受人大政协监督，办理人大议案1件、建议93件和政协提案144件，办复率100%。认真吸纳社会各界意见建议，民主党派、工商联围绕经济振兴、社会进步、民生改善积极建言献策，提出许多真知灼见。推进政府信息公开，市政府门户网站访问量超过4600万人次。市民热线受理满意率92.1%。廉政建设进一步加强。全面落实政府系统党风廉政建设“两个责任”，推进正风肃纪，落实审计整改事项208项，查处“为官不为”行为136起、处分134人。

　　各位代表！以上成绩来之不易，值得珍惜！这是省委、省政府重视湛江、关心湛江的结果，是市委坚强领导、科学决策、敢于担当、干在实处的结果，是市人大、市政协和社会各界关心支持、鼎力相助的结果，是全市人民迎难而上、奋力拼搏的结果。我谨代表市人民政府，向全市人民，向人大代表、政协委员，向各民主党派、工商联、人民团体及社会各界人士，向中央、省驻湛单位，向驻湛人民解放军、武警官兵，向公安政法干警，向所有支持、参与湛江发展的老领导、老同志和港澳台同胞、国际友人，向为重点项目、重大工程建设“舍小家顾大家”、作出特殊贡献的征地拆迁群众，表示衷心的感谢，致以崇高的敬意！

　　回顾工作，我们信心满怀；面对问题，我们亟待破解：一是发展质量不高、效益欠佳，经济总量、人均水平与中心城市地位不匹配。二是产业结构不合理，产业发展不配套、链条不长、集聚度不高，园区土地储备不足，产业发展严重受限，规上工业企业数量偏少、实力偏弱。三是高端人才紧缺，科技成果转化率不高，创新驱动发展的基础不牢。四是主城区组团分散、中心不突出，支撑城市发展的产业引擎不强，规划建设管理相对粗放，城市功能配套不足，公共服务不优。五是区域发展不均衡，县域城镇化、工业化水平偏低。六是社会民生欠账较多，精准扶贫任务繁重，市民就医、出行、住房、上学等问题仍较突出。七是政务服务仍存在水平低、效率不高等问题；公务员队伍敢担当、抓落实、促发展的意愿不够、能力不足；个别部门推诿扯皮、敷衍塞责，慢作为、不作为、乱作为，消极腐败现象时有发生。对此，我们必须高度重视，坚持问题导向、目标导向和责任导向，以人民为中心，以更大格局、更高站位谋划，推动湛江发展，走高质量效益型发展之路，加快建设中心城市，努力成为广东新的增长极。

　　进入新时代，湛江正在成为实施国家和省发展战略的重要支撑，迎来千载难逢的发展机遇。湛江的机遇在于国家深入推进“一带一路”建设，作为我国与东盟国家经贸合作最前沿，我们可以加快“走出去”“引进来”，在更广范围、更深层次扩大对外开放，全面参与国际经济合作；在于国家加快建设海洋强国，作为海洋大市，我们可以进一步优化海洋经济结构，提升海洋产业竞争力，实现蓝色崛起；在于国家实施军民融合发展战略，作为驻军大市，我们可以加快发展军民融合产业，推进高科技产业发展；在于省委省政府构建更加有效的区域协调发展机制，推进粤东西北振兴发展，把东、西两翼打造成为广东新的增长极，我们可以有效利用省的政策、资金，有效利用珠三角先进产业资源，通过产业共建在更高起点加快发展。

　　进入新时代，湛江发展最根本的机遇，在于我国经济已由高速增长阶段转向高质量发展阶段。今日湛江，有较好的发展基础，没有沉重的过剩产能和库存；有富集的土地、海洋资源，没有超负荷的投资开发强度；有良好的自然生态，没有沉重的环境治理包袱。进入新时代，迈上新征程，我们不用沿袭“先污染后治理”“边污染边治理”的传统发展模式，不用重走低质低效的粗放发展老路，我们可以利用新一轮科技革命和产业革命的成果，改造升级存量，引进优质增量，走高质量效益型发展之路，绘制“最美的发展图画”。

　　推动高质量发展，是发展机遇，更是发展大势所趋、发展规律使然。坚持高质量效益型发展，是湛江落实习近平新时代中国特色社会主义经济思想的具体实践，是推进落实“三大抓手”实施方案和“五大产业发展计划”，确保全面建成小康社会，加快建设北部湾中心城市和省域副中心城市，打造广东新的增长极的必由之路。根据中央、省委、省政府和市委的统一部署，围绕推动高质量发展，市政府将坚持以习近平新时代中国特色社会主义经济思想统领经济工作，坚持质量第一、效益优先，坚持以供给侧结构性改革为主线，推动发展质量变革、效率变革、动力变革，推动质量与效益贯穿经济社会发展全过程，确保湛江经济实现有质量有效益的中高速增长。一是突出实干争先，面对湛江发展不足的现实，面对稍纵即逝的政策机遇，牢记发展就是硬道理，牢记“时间就是金钱、效率就是生命”，只争朝夕，崇尚实干，抢抓机遇拼发展。二是突出基础长远，高标准加强水利、铁路、公路、水运、航空、管道、信息、物流等基础设施网络建设，高质量实施机场迁建、高铁、高速公路和港口重点建设项目，坚持互联互通、无缝衔接，优化站场规划设计，加快建设面向未来的现代化基础设施。三是突出扬长补短，推动互联网、大数据、人工智能与实体经济深度融合，推动实体经济与科技创新、现代金融、人力资源协同发展，立足深水大港口，引进优质大项目，发展先进大产业，重点发展钢铁、石化、船舶、海工装备先进制造业，推动形成“港产城”融合发展生态圈，建设世界级绿色高端沿海临港重化产业基地。四是突出精明增长，立足拥湾建城、以港兴市的城市成长规律，立足碧海蓝天的优良生态，拓展国际视野，对标国内一流，推动城市发展由外延扩张转向高质量发展，加快新型城镇化步伐，建设高品质现代化城市。五是突出人民至上，秉持尽力而为、量力而行，下大力气补短板、强弱项、促公平，最大限度增加优质民生供给。六是突出需求导向，聚焦市民和企业需求，坚持把优质服务人民、有效保障企业作为政府工作的努力方向，转变政府职能，深化“放管服”改革，创新服务方式，建设人民满意的服务型政府。

2018年工作安排

　　2018年是贯彻党的十九大精神的开局之年，是改革开放40周年，是决胜全面建成小康社会、实施“十三五”规划承上启下的关键一年。

　　今年市政府工作的总体思路：全面贯彻党的十九大精神，坚持以习近平新时代中国特色社会主义思想为统领，认真落实中央经济工作会议和省委十二届二次、三次全会精神，全力落实市委十一届三次、四次全会精神，坚持以人民为中心，坚持稳中求进工作总基调，坚持新发展理念，坚持以供给侧结构性改革为主线，坚持高质量发展，推进交通、产业和城市现代化，大力实施乡村振兴战略，统筹推进稳增长、促改革、调结构、惠民生各项工作，打好防范化解重大风险、精准脱贫、污染防治攻坚战，引导和稳定市场预期，加强和改善社会民生，促进经济社会实现高质量效益型发展，推进全面建成小康社会，加快建设北部湾中心城市和省域副中心城市，打造广东新的增长极。

　　今年经济社会发展的预期目标：生产总值增长7.5%左右，实际工作按8%左右争取；规上工业增加值增长9%左右；固定资产投资和社会消费品零售总额增长10%左右；外贸进出口总额增长7%；实际利用外资增长3.5%；港口吞吐量达到3亿吨；一般公共预算收入剔除一次性因素，可比增长10%；居民人均可支配收入增长7.5%左右；消费价格涨幅控制在3%以内；城镇登记失业率控制在3.5%以内；单位生产总值能耗下降3.5%，完成省下达节能减排降碳目标任务。

　　实现上述目标，今年重点抓好以下工作。

　　（一）全面推进交通现代化，加快构建快速立体交通网络体系。坚持交通先行，坚持在交通强国战略中主动作为、后发追赶、赢得先机，加快实施一批面向未来的现代化交通基础设施项目，推动高铁进城、高速入市、无缝换乘、快速对接，早日建成全国性综合交通枢纽。年内实施国际机场、高速铁路等37个重点交通项目，完成投资206亿元。

　　全面实施机场迁建大会战。按时完成征地拆迁任务，按时交付建设用地，落实用电、给排水施工，保障机场航站楼等工程全面施工；加快安置区建设，开工建设空港大道、机场大道和换乘中心等配套设施。

　　全面实施铁路建设大会战。编制完成湛江枢纽总图规划；建成高铁西站客运综合交通枢纽，完成茂湛铁路电气化改造，确保6月深湛高铁顺利通车，争取开行到达北京、上海和珠三角核心城市的直通车；落实高铁中心城区枢纽站选址，建设高铁机场站；建成东海岛铁路客货共线和宝钢工厂站；开工建设东海岛、宝满港区铁路支线，规划建设粤海铁路乌石支线，推进合湛高铁建设；加快推进广湛客专和张海、湛海高铁前期工作，力争获得国家批准。

　　全面实施公路建设大会战。压实责任、倒排工期，确保湛江大道3月完成征拆、4月开工建设；建成西城快线，加快海川大道扩建和调顺跨海大桥建设，确保城市出入口更加顺畅；开工建设环城高速南三岛大桥、茂湛高速改扩建，以及广东滨海旅游公路徐闻示范段和吴川、坡头先行段等项目；动工湛廉快线、疏港大道二期等9项国省道升级改造项目；全线建设汕湛高速吴川支线，加快建设东雷、玉湛高速，规划建设湛徐高速横向支线。

　　全面实施港口建设大会战。落实与招商局集团“1+N”战略合作协议，设立“港产城”融合发展基金，保障宝满港区集装箱一期扩建等20个合作项目全面落地。动工30万吨级航道改扩建等6个项目，疏通亚士德航道，推进建设邮轮码头，加快建设中科炼化配套、霞山港区通用和东海岛港区液化码头。建成徐闻港区南山作业区客货滚装码头、徐闻港进港公路和湛徐高速徐闻港支线“三大工程”，动工建设徐闻港区南山作业区客运大楼。

　　（二）全面构建新型产业体系，高质量发展实体经济。注重吸收先进理念，引入先进工艺，借鉴先进模式，加快营造一流营商环境，配套一流基础设施，建设一流产业园区，培育一流企业品牌，以“五大产业发展计划”为着力点，推动实体经济高质量发展，推动产业体系迈向中高端。

　　高质量发展新型产业。致力打造精品工程、塑造质量品牌，积极发展现代钢铁、生物制药、先进海工装备、先进船舶制造、数字印刷、精品包装等先进制造业，支持军民融合、精细化工、现代物流、新能源、新材料等重大产业项目加快建设，全力争取纳入珠江西岸先进装备制造产业带，推进“港产城”融合发展，建设世界级绿色高端沿海临港重化产业基地。实施雷州半岛25万吨生态海盐等34项现代产业项目。支持湛江钢铁基地稳定生产、优化质量、拓展品种、削减成本，以智慧制造打造数字化钢铁；通过产能置换，启动二期3号高炉系统建设前期工作，提升钢铁产业竞争力。支持中科炼化建设，完成工艺装置钢结构安装，年内完成投资100亿元左右。加快乌石17－2油田群开发。支持湛江卷烟厂等企业优化产品结构，实现提档升级。

　　高水平建设一流产业园区。优化园区规划。坚持国际视野、国内一流，编制完成产业园区发展规划，落实空港经济区、临港工业园、高新产业园、都市型工业园和高铁经济带规划布局。夯实园区公共服务平台，提升园区配套设施，加快建设产业园区与高速公路之间的连接线。提升开发水平。重视盘活园区存量建设用地，支持现有企业追加投资、升级改造，提高土地利用强度；优化园区准入条件，对投资强度高、税收贡献大、带动能力强、社会效益好的企业，给予用地指标、用地取得、供地方式、土地价格等方面优惠支持。强化征地拆迁和土地供应，12个省级产业园新增供应土地5%以上。引进优质企业。组建市招商机构，落实产业招商地图，落实“延链补链”产业招商，全力以赴引进优质增量。坚持面向大型央企、民企和珠三角先进制造业企业，全力支持广大湛江乡贤回归，培育发展高质量效益型产业。发展主导产业。支持央企和有实力的外企合作共建石化产业园区，大力发展精细化工，延伸石化产业链条，支持钢铁配套园区开发生产钢结构装配式建筑产品，加快湛江产业转移园扩能增效。完善麻章森工、太和工业园以及海田物流产业园配套设施；推动湛江临港工业园发展现代临港产业；支持奋勇高新区建设东盟产业园，发展军民融合产业；支持南三滨海旅游示范区建设军民融合智慧海洋产业园。

　　高起点发展现代服务业。加快服务业升级发展，高起点规划建设省现代服务业次中心，培育发展总部经济、信息经济和服务经济，推动传统服务业向现代服务业升级。支持实体零售企业加快线上线下融合，建设电商示范企业。规划建设一批商贸综合体、高端购物商城，提高共享经济、农村电商发展水平。推进公共文化体育服务均等化，培育智慧居家养老服务。引进高层次展会，办好2018中国海博会、东盟·农博会、中国水博会，壮大会展经济。培育发展数字经济，出台数字经济发展规划，推进5G技术应用，构建高速、移动、安全、泛在的新一代“信息高速公路”。普及4G使用，全方位实施“互联网+”行动计划，引进龙头企业发展云计算和大数据产业，打造数字湛江，建设智慧城市。支持湛江港实施“五大战略”，打造华南铁矿石分销中心、华南石油集散中心、中西部粮食化肥物流中心、千亿元大宗商品交易中心、区域性集装箱运输中心及保税物流中心，实现集装箱吞吐量突破100万标箱。坚持以广东滨海旅游公路湛江段沿线为重点，支持有实力的优质企业高水平开发雷州半岛滨海、人文、生态旅游资源，扎实推进景区创A升级，建设广东全域旅游示范区。加快建立多主体供应、多渠道保障、租购并举的住房制度，支持兴建租赁公寓，开拓住房租赁市场，鼓励长期租赁，坚决打击违法租赁、违规炒作、囤积房源和非法中介机构，推广现房销售，探索建立共有产权住房制度，强化市场监管，合理引导预期，确保房地产市场稳定健康有序发展。

　　高强度推进有效投资。安排中科炼化等182项重点项目，年度计划投资430亿元，其中省重点项目年度计划投资362亿元。落实项目储备。高效推进项目申报对接，确保湛江钢铁基地3号高炉、南海西部天然气管道登陆等更多项目列入国家和省专项规划、年度计划和重大项目库，通过项目最大程度获取上级政策、资金支持。落实项目进度。下决心优化用地、规划、立项等前置审批流程，提高项目前期工作效率，推动更多企业投资项目建设直接落地。加强项目建设跟踪督查，确保湛江大道等69个合作项目、湛江港集团增资扩股等84个重大签约项目快落地快动工。坚持每季度集中签约一批项目、每季度第一个月集中开工一批项目，形成“开工一批、建设一批、竣工一批、谋划一批”项目建设局面。落实要素保障。出台重大产业链项目落地指导意见，优化用地供应方式，推行土地弹性年期出让，鼓励企业长期租赁、先租后让、租让结合使用土地，推动高效快速供地。

　　高标准优质服务企业。强化企业发展经济、创造财富的主体地位，聚焦企业需求，对接国际通行规则、对标国内先进一流，切实为企业生产经营提供高质量高效率服务。强化政策落地。用好用足国家和省系列政策，进一步清理规范行政事业性收费，实行工伤保险浮动费率，全力落实降低制造业企业成本、支持实体经济发展43条，最大限度降低企业成本。强化机制保障。建立服务企业快速响应与企业服务评价机制，落实“一企一策”“一事一议”精准服务，推进“暖企”行动常态化、机制化，年内解决工业企业重大历史遗留问题40个。强化氛围营造。发布年度工业百强与纳税百强企业，推行政府资源配置与企业税收贡献挂钩，尊重和支持企业发展壮大，激发和保护企业家精神，鼓励更多实体企业投身创新创业。

　　（三）全面推进新型城镇化，加快建设高品质现代化城市。立足拥湾建城、因港兴市发展特色，坚持高起点规划、高水平建设、高标准管理，加快推进城市现代化。

　　推动城市高质量发展。彰显海湾城市特色推进主城区强芯提质。落实“多规合一”，立足2035年，展望2050年，组织编制新时代新一轮城市总体规划；强化规划约束，加强湛江湾立法保护和雷州半岛滨海地区规划建设管控，依法开发城区港口岸线；开展城市设计，严格控制一湾两岸建筑高度，加快形成错落有致、张弛有度、富有韵律的城市天际轮廓线，打造层次丰富的滨海景观；有效保护历史街区、历史建筑、传统村落和南粤古驿道，彰显城市底蕴、地域特色与时代风貌。优化空间布局，完善赤坎、霞山、开发区主城区功能，规划建设一批大型商业综合体、高端写字楼，发展都市型经济。升级城区市政配套设施，改善道路微循环，新建南岑路、二环路，建成盛邦路、银帆路、上坡路。加快城市更新步伐，申报国家“城市双修”试点，实施赤坎旧大天然片区、中海油南海西部矿区和霞山东新路等15个“三旧”改造项目。建设市区引水工程，启动坡头城区供水工程。注重以业兴城推进新区开发。重视培育新区发展引擎，落实海东高新区规划布局，建设海洋生物医药产业园，建设南方药都。动工建设地下综合管廊一期，完善新区公共配套设施，加快推进东旺大道等13个项目建设。完善麻章区城市功能，高标准谋划西城新区开发，严格管控高铁西站周边及湛江大道沿线土地；补齐市政配套设施短板，推进疏港大道、湖光快线改扩建，争取动工建设西城第一污水处理厂；开工建设深国际·湛江综合物流港项目，打造北部湾现代物流高地。坚持用绣花精神治理城市。加快建设交通大数据决策平台，充分利用大数据支撑，提升城市规划建设和城市治理水平。大力推进城市美化、绿化、亮化，落实园林绿化三年行动计划，加快市区道路林荫路改造，升级改造森林公园，建设三岭山-湖光岩景区绿道，创建国家生态园林城市；启动海滨大道、人民大道、椹川大道三条主干道和机场路亮化工程；集中整治“六乱”，扎实推进城市精细化管理，全力加快市容市貌提档升级。规划建设城中村与城乡结合部公厕，扎实推进城乡厕所革命。

　　加快县域新型城镇化、工业化。支持做大做强县域工业。坚持工业兴县、特色强县，支持县域产业园区扩能增效，推动县域重点工业项目加快建设，支持县域工业加大技改投入，延伸产业链条，强化品牌培育，壮大特色产业集群，补齐工业发展短板。支持做大做强县城和重点镇。支持高起点修编完善县城总体规划，重点补齐交通、环保、电力、信息基础设施短板，完善教育、医疗、商业、文体等公共服务设施。支持徐闻县充分利用海南国际旅游岛的外溢辐射效应，对琼州海峡北岸实施高水平谋划、高质量开发、高效益发展。支持安铺、南三、麻章等特色小镇彰显个性、差别发展，打造美丽小镇。

　　（四）全面实施乡村振兴战略，推动农业农村优先发展。坚持“产业兴旺、生态宜居、乡风文明、治理有效、生活富裕”，整合涉农资金，支持农业综合发展，市级财政“三农”投入翻番，新增投入5亿元。加快落实我市乡村振兴战略实施方案，统筹开展“七大行动”，推进实施雷州半岛现代农业、水利设施规划，争当全省乡村振兴排头兵。大力发展现代农业。深化农业供给侧结构性改革，落实9项重点改革任务。坚持用现代农业的理念改造提升传统农业，大力推广种植、养殖新技术和新品种，落实粮食安全负责制，建设水稻、甘蔗全程农机作业试点样板，复耕水田2.45万亩，建成33.7万吨新粮仓。坚持用开放思维发展现代农业，积极引进现代大型农业龙头企业，引进农业高新技术，发展农业优势特色产业，构建全产业链，提高农产品精深加工和副产品综合利用水平。支持徐闻创建粤西首个省级农产品加工园区，加快建设北部湾农产品仓储流通综合示范园等区域性农产品物流中心。发展农村普惠金融，支持农垦和中国热科院所属研究机构农业科技成果就地转化，推进“互联网+现代农业”，推动湛江农海特产品网上行，推动小农户与现代农业有机衔接，促进农村一二三产业融合发展。积极促进农民增收。盘活农业农村资产、资源，推动农村集体经营性建设用地入市，完善农村土地“三权分置”制度，完成农村土地确权登记颁证。开展省级农村集体产权制度改革遂溪试点。加快建设美丽乡村。落实省定贫困村创建新农村示范村总体方案和农村生活垃圾处理条例，全面开展218个省定贫困村“三清理、三拆除、三整治”。绿化美化百条乡村，实现镇（街）村环卫作业服务市场化全覆盖。加大农村基础设施建设投入，完成雷州半岛水利规划年度建设项目；认真落实“四好”要求，建好管好护好运营好农村公路，完成县道路面改造收尾，升级改造乡道100公里。开展首批20条特色乡村创建试点。

　　（五）全面推进创新驱动发展，加快建设创新型城市。坚持把创新作为引领高质量效益型发展的第一动力，完善企业为主体、市场为导向、产学研深度融合的创新体系，着力培育发展新动能。

　　支持高新技术产业做优做强。支持企业与高校、科研院所共建产业创新联盟，推动大型骨干企业研发机构全覆盖，力争高新技术企业超过160家。加快科技成果转化。出台促进科技成果转化实施办法，建设成果转化平台，完善转化服务链条，争取技术合同交易额突破1亿元。推动军地、校地、垦地深度融合，支持驻湛高校、科研院所科技成果就地转化。加快建设新型研发机构，年内各类研发机构达到280家。落实人才引进资助政策。规划布局国际学校、人才公寓，完善人才、住房、医疗及大企业服务等优惠政策，实施人才“十百千”等七大人才计划，培育精益求精的“港城工匠”，鼓励紧贴用户需求的“微创新”，培育开拓进取的科技企业家，强化科技创新的人才和智力支撑。

　　推进传统产业转型升级。争取更多项目、产业园区扶持和技术改造资金，支持企业引进先进的设计理念、先进的技术工艺和先进的生产模式，开展关键技术攻关，全面推进传统企业装备智能化、产品高端化、管理现代化。推动渤海粮油等7家省级“两化融合”管理体系“贯标”试点企业通过评定。健全智能家电研发体系，支持龙头家电企业拥有自主知识产权的发明专利，培育壮大自主品牌，申报智能制造试点示范项目和智能制造标杆企业；提升医药、羽绒、家具、糖业、食品、水海产品等产业集群优质发展水平。支持湛江发电厂等企业增加生产计划，实现扩产增效。完善中小微企业综合服务，用活省市互保资金池、小微贷等金融工具，支持企业“个转企、小升规、规改股、股上市”，力争新增120家规上工业企业、1－2家上市公司。加快建设质量强市，落实“三品”战略和商标品牌战略，培育发展本土品牌。

　　加大陆海统筹推进蓝色崛起。落实省沿海经济带综合发展规划和市蓝色海洋综合开发计划，全力拓展蓝色发展空间。加快实施南海海洋生物医药资源公共服务平台等21个海洋创新项目，推进建设海洋经济创新发展示范城市。加快传统捕捞养殖业转型升级，建设徐闻前山对虾种苗和东海岛西南部深水网箱养殖基地，建成流沙珍珠孵化养殖加工一体化基地，打造海洋牧场、蓝色粮仓。加快建设6个省级现代渔港和两个国家一级渔港。

　　（六）全面深化改革、扩大开放，加快提升城市国际化水平。坚持全球化视野、国际化思维、法治化制度、本土化特色相结合，着力推动改革引向深入，着力形成全面开放新格局。

　　扎实推进重点领域和关键环节改革。启动实施一批改革事项，改革国有资本授权经营体制，加快资源资产划拨划转，组建资本投资运营公司，推进存量项目PPP改造，完成基投集团、交投集团融资目标，推动国有资本做大做强做优；深化国企改革，完成汽运集团、湛化集团等市属企业改制，推进“僵尸企业”出清重组、有序退出，发展混合所有制经济，支持民营企业发展壮大。完善地方财政体制，深挖财政统筹潜力，健全预算制度，优化财政支出结构，增强公共财政支出对稳定经济增长、对促进农业和教育优先发展、对改善社会民生的支撑作用。加强金融监管与风险处置，支持各类企业按改制规定入股参与农信社改制，组建农村商业银行，打造直接服务“三农”的地方金融机构；完善县级综合征信中心。深化户籍制度改革，推动非户籍人口落户城市。完善城市综合管理和执法体制，稳妥推进环保监测垂直管理和事业单位分类改革。深化司法体制综合配套改革。

　　深化“放管服”改革打造一流营商环境。坚持以简政放权为重点，推进审批经济向服务经济转变，推进政府服务提质增效，全力营造北部湾城市群服务效率最高、管理最规范、综合成本最低的国际化法治化营商环境。围绕增创营商环境优势，坚持对标先进，发达地区已经先行的，我们必须借鉴；兄弟城市已经推行的，我们必须做到、而且必须做到更好；各级各部门必须从局长抓起，从科长抓起，从科员抓起，全员构建亲清政商关系，全员参与建设营商环境。坚持“能转尽转、能放尽放”，加快建设行政审批“中介超市”，全面推行行政审批委托制，继续削减和委托下放一批市级行政职权事项，最大限度向基层简政放权，同步落实基层事权交接，确保放得下、接得住、用得好。坚持改革投资建设工程项目审批制度，确保审批时限从219个工作日压减至32个工作日，审批事项不超过15个；全力承接省政府委托我市建设用地审批工作。坚持实施市场准入负面清单管理，清理废除妨碍统一市场和公平竞争的各种规定和做法，大幅放宽市场准入，复制自贸区“证照分离”试点经验，注重用透明的法治环境稳定市场主体预期。坚持推进商事登记后置审批事项分类改革，全面推行“证照分离”，着力破解“办照易办证难”；推行全程电子化登记，推广商事登记银政直通车服务，落实“多证合一”“减证便民”，全面激发“众创”深厚潜力。坚持创新监管方式，推进市场监管综合执法，推行“双随机一公开”监管、“一库两平台”信用协同监管和“互联网+”智能监管，提高市场监管现代化水平。

　　加快形成全面开放新格局。全力拓展对外经济合作新模式、新路径、新体制，坚持在更大范围、更广领域、更高层次推动双向开放。对内主动融入全省和北部湾城市群一体化发展。主动对接珠三角，融入粤港澳大湾区，争取与珠海、佛山和深圳南山共建产业合作区，发展“飞地经济”。积极对接海南岛、联动北部湾，参与编制北部湾城市群综合交通运输规划，推动港航资源整合，加快琼州海峡一体化发展，共建琼州海峡经济带和湛江—北海粤桂经济合作区。与绥化市、柳州市开展对口合作，在协同发展中发挥湛江优势、展现湛江作为、做出湛江贡献。对外构建以“一带一路”为重点的开放新格局。深化与东盟、欧盟和南亚、中东、非洲的经贸、科技、人文合作，支持蔗糖、饲料、农海产品、家电和建筑业利用国际资源与市场做优做强。借力招商局集团海外布局，开辟更多集装箱国际航线，提升“一带一路”海上合作战略支点城市建设水平。优进优出稳定外贸增长。支持外贸企业用足出口退税、信保和便利通关政策，发展跨境电商、冷链物流和供应链金融，全力拓展“一带一路”沿线新兴市场，扩大外贸出口。推广国际贸易“单一窗口”标准版，推动口岸“三互”大通关。加快跨境电商网购保税进口，加快保税业务向国际物流配送、国际中转、跨境电商转型，探索建设自由贸易港。

　　（七）全面增加优质民生供给，努力提升群众生活质量。坚持提标兜底同步，加快补齐民生短板，努力提供更多优质民生服务。

　　尊重民意办好民生实事。办成十件民生实事：城乡居民基础养老金每人每年增加120元；城乡居民医保各级财政补助标准每人提高到480元；人均基本公共卫生服务经费达到55元；建成保障房960套；新增市区义务教育学位3700个、学前教育学位1.5万个；完成市区293条小街小巷路灯安装，实现全覆盖；完成740公里精准扶贫农村公路硬底化；解决农村2290条自然村102万人安全饮水问题；建成350个村级污水处理设施；建成500间村级公建民营卫生站。统筹办好民生微实事。民生无小事，建立民生微实事项目库，坚持市级统筹、县（市、区）为主体，建立市、县（市、区）、镇（街）、村（居）四级协作联动机制，快速解决群众身边的小事、急事、难事和烦心事，切实增强人民群众的获得感、幸福感。

　　有效加强民生保障。多层次提供公共服务，全力支持高校毕业生、农民工等重点群体多渠道就业创业，新增城镇就业7.5万人、失业人员再就业3万人，扶持超过2500人创业就业。落实全民参保计划，城乡居民养老保险参保率超过99%；落实企业职工养老保险扩面征缴，推进公务员、参公人员参加工伤保险。完善城乡社会救助体系，提高低保、特困人员供养和医疗救助水平，关爱留守儿童，确保困难群众求助有门、受助及时。

　　坚决打赢精准脱贫攻坚战。坚持把精准脱贫攻坚作为第一民生，加强督查考核，压实主体责任，强化产业扶贫，集中一切可以集中的资源、调动一切可以调动的力量精准扶贫，确保扶贫增收项目快落地快实施快见效，促进贫困户有效增收。注重扶贫同扶志、扶智相结合，激发脱贫内生动力，保证完成年度精准脱贫任务。

　　繁荣发展社会事业。坚持教育优先发展。市级财政增加教育投入30%以上，加快建设广东教育现代化先进市，发展优质公平、人民满意的教育。深化教育综合改革，大力建设公办、普惠性民办幼儿园，推动学前教育扩容提质；落实“三年行动计划”，支持和规范社会力量办学，制定新建住宅小区配建标准化学校、无偿移交政府办学实施办法，加快建设湛江一中和岭师附中新校区，加快解决中心城区学位紧缺和“择校热”“大班额”问题；落实第二期特殊教育提升计划；加强教职工编制统筹、跨区域调整和动态管理，落实乡村教师“两相当”和农村教师生活补助，完成农村教师免费轮训。实现全市中小学100%宽带接入，推进教育信息化、现代化。支持广东海洋大学建设高水平学科，推进广东医科大学、岭南师范学院新校区建设，支持广东文理职业学院“专升本”；深化产教融合、校企合作，提升职教发展水平。推进实施健康湛江战略。围绕增加优质医疗卫生服务供给，全面深化医药卫生体制综合改革，推动二级以上公立医院参与医联体建设，实施公立医疗机构药品和医用耗材配送改革，落实药品集中采购第三方交易“两票制”;加快建设广东医科大学附属医院海东院区，开工建设市妇幼保健院新院、公共卫生医院和第二中医院综合住院大楼，启动市第一中医院搬迁改造，升级建设18家县级公立医院，启动建设5县（市）120急救指挥中心，基本完成7家中心卫生院主体工程；建成区域全民健康信息综合管理大平台，加快卫计工作信息化。推动文体事业蓬勃发展。引进优质文化主体，启动湛江市图书馆改扩建，推动全民阅读，打造书香湛江；完成村（社区）基层综合文化服务中心全覆盖，推进县（市、区）图书馆、文化馆、博物馆全覆盖；积极筹建社区剧院，丰富群众文化生活；引进优质演艺团队，盘活湛江影剧院，发展文化产业，活跃文化市场。完成自然村落历史人文普查和百部地方志丛书编修，完善非遗记录工程。推动县级国家综合档案馆建设达标。广泛开展全民健身，举办半岛骑行等系列健身活动，办好中网青少年巡回赛、南粤古驿道定向大赛等活动。积极备战第15届省运会和第8届省残运会。完成第四次经济普查。做好妇女儿童、残疾人、民族宗教、外事侨务、海防打私、人防、气象、地震等工作。

　　打造共建共治共享社会治理格局。全力落实省委、省政府“把广东建成全国最安全稳定的地区”要求，加快完善社会治理制度，提升社会治理社会化、法治化、智能化和专业化水平。深化平安湛江建设，保持严打整治高压态势，重拳打击黄赌毒黑拐骗等突出违法犯罪，打击电信诈骗、网上虚假信息诈骗等新型犯罪，排查整治治安重点地区和突出治安问题。深入推进全民禁毒工程，强力压减毒品犯罪，确保雷州禁毒重点地区整治顺利“摘帽”，筑起湛江禁毒“钢铁长城”。实施“雪亮工程”，实现治安视频监控零死角、全覆盖，全面构建社会治安防控体系。全面落实社会治理重心向基层下移，推进基层派出所与基层平安建设融合，开展基层民主协商和农村社区建设试点，实现政府治理和社会调节、居民自治良性互动。深入开展信访积案大起底，健全社会矛盾排查预警和调处化解机制。深入开展“七五”普法教育。坚持安全发展理念，压实安全生产责任，落实重大安全隐患整改和重点行业专项整治，坚决遏制重特大事故发生。落实食品药品安全监管要求，强化“从田间到餐桌”全程监管，推进消费维权共治，坚守“舌尖上的安全”。加强应急演练，提升突发事件防范处置和防灾减灾救灾能力。

　　（八）全面打造优质生态环境，实现发展与美丽双赢。生态是湛江的优势，必须践行“绿水青山就是金山银山”，巩固提升城乡环境质量，悉心呵护生态文明湛江名片。

　　扎实推进绿色发展。落实投资节能评估审查，开展第二次污染源普查和第三次国土调查，推进资源节约和循环利用，完成年度能耗下降和控制目标。深化循环化改造试点，加快建设国家循环经济示范市。有序发展清洁能源产业，发展绿色建筑与钢结构装配式建筑。倡导简约适度、绿色低碳生活方式，开展创建节约型机关、绿色家庭、绿色学校、绿色社区和绿色出行等行动。

　　坚决打赢大气、水、土壤污染防治“三大战役”。全力保护湛江的一片蓝天、一湾清水、一方净土。落实中央、省环保督察整改，按时完成红树林、饮用水源保护区和城市石化企业管控等11项整改工作。落实我市大气环境质量持续达标30条，深化工业污染综合防治，全面防治移动污染，有效治理挥发性有机物，坚决防治扬尘污染，严格整治露天焚烧，确保空气质量保持全省前列。落实“水十条”，全面推行“河长制”，形成责任明确、协调有序、监管严格、保护有力的河流管理保护机制，有效强化源头防治，强化黑臭水体和重污染河流治理，强化水生态修复，强化依法监管，严惩企业偷排行为，重点整治菉塘河、南柳河等城区22条河涌黑臭水体，加大力度综合治理九洲江－鹤地水库、小东江和南渡河流域水环境。落实“土十条”，详查土壤污染，防治重金属污染。加强固体废物环境管理，改造东纯村建筑垃圾处置场，建设3个县城生活垃圾焚烧厂。

　　切实提升生态创建水平。落实主体功能区规划，开展生态红线、县域“三区三线”空间划定工作。完成海岸线调查，健全海岸线管控机制，强化79段海岸线分段分类管控；完成湛江湾（北部）等5个海岸带整治修复，推进3个人工鱼礁和乌石、特呈岛国家海洋公园建设。落实国家海洋督察整改任务，有效遏制盲目开发、破坏海岸带行为，坚决保护美丽海湾。扎实推进新一轮绿化湛江大行动和雷州半岛生态修复，计划造林更新6.5万亩，绿化美化乡村100条。实施林业“四大”重点生态工程，构建宜居宜行绿色生态网络。强化“创文”工作，提升“巩卫”成果。

　　（九）全面落实从严治政，建设人民满意高效政府。全面推进政府政治、思想、作风、纪律建设，建设忠诚干净担当的高素质专业化公务员队伍。

　　旗帜鲜明讲政治。坚持把政治建设摆在政府建设的首要位置，坚持用习近平新时代中国特色社会主义思想指引政府工作，强化“四个意识”，坚定“四个自信”，坚定维护以习近平同志为核心的党中央权威和集中统一领导，自觉在思想上政治上行动上同以习近平同志为核心的党中央保持高度一致，确保政府工作始终沿着正确方向前进。严守政治纪律和政治规矩，坚决落实党中央国务院、省委省政府和市委决策部署，保证政令畅通、令行禁止。推进政府系统“大学习、深调研、真落实”行动，推进“两学一做”学习教育常态化制度化，认真开展“不忘初心、牢记使命”主题教育。

　　牢记宗旨优服务。坚持问政问计于民，切实转变政府作风，深入调查研究下功夫解难题，始终保持同人民群众的血肉联系，保持对人民的赤子之心，全力推动工作重心下移，扑下身子深入群众，面对面、心贴心、实打实做好群众工作。提升12345市民服务热线、在线访谈等民意平台综合服务水平，健全发布、解读、回应“三位一体”政务公开体系，建设150台“市民之窗”自助服务终端。坚持市级统筹、整体联动、部门协同、一网通办，基本建成“互联网+政务”体系，加快建设“数字政府”。

　　依法行政重规范。坚持民主集中制，凡属“三重一大”事项一律按照政府议事规则研究决定。坚持依法履职、服务人民，加快建设法治政府。持续推进规范性文件清理，深入推进行政执法规范化、信息化，开展行政执法专项监督检查，做好行政复议、应诉工作，推进严格规范公正文明执法。认真执行重大行政决策程序规定，强化决策后评价监督。自觉接受人大和政协监督，定期向人大报告工作、向政协通报情况，认真倾听民主党派、工商联和社会各界人士意见，全力办好建议、提案。

　　加强廉政硬约束。坚持把落实管党治党政治责任作为最根本的政治担当，高标准推进政府系统党风廉政建设。严格执行纪律，强化政治纪律和组织纪律，严肃廉洁纪律、群众纪律、工作纪律、生活纪律。锲而不舍落实中央八项规定精神，继续在常和长、严和实、深和细上下功夫，驰而不息整治“四风”，密切关注享乐主义、奢靡之风新动向新表现，纠正形式主义、官僚主义，坚决整改表态多调门高、行动少落实差等问题，政府系统各级领导要自觉“对焦画像”，以“头雁效应”带动政风全面好转。坚持节用裕民，严控“三公”经费支出。强化权力运行监督，推进审计监督全覆盖，加强审计结果利用。严查各类违纪违法案件，严厉整治群众身边的不正之风和腐败问题。全体公务员要知敬畏、存戒惧、守底线，习惯在接受监督和约束的环境中工作生活。

　　强化担当重实干。强化责任落实。任何人任何部门在责任面前不得以任何理由、任何借口推诿扯皮、敷衍塞责，必须增强真抓的实劲、敢抓的狠劲、善抓的巧劲、常抓的韧劲，勇挑最重担子，敢啃最硬骨头，善接最烫山芋，抓铁有痕、踏石有印，把所负责的工作抓紧抓实；坚决打破空间距离、条块分割、行政区划，加强协同攻坚，加强政策配套，加强督查落实，确保工作措施落地生根、开花结果。强化效率提升。推进政府工作项目化、项目目标化、目标责任化、责任考评化，全面推行政府工作定主体、定时限、定责任，落实重点项目建设挂图作战、跟踪进度、监管到位，切实把任务进度细化到每月每周每天，加强重点项目、重要工作、重点事项、重大决策督查督办，高效率、快节奏推进政府工作。强化严明奖惩。扎实推进“四个全面”绩效考核，用好“两函两书”督查利器，着力整治“为官不为”。健全激励和容错纠错机制，旗帜鲜明为敢于担当、踏实做事、不谋私利的干部撑腰鼓劲。全体公务员要拜人民为师，向人民学习，努力增强“八大本领”、做到“五个过硬”，放下架子，扑下身子，察实情、出实招、办实事、求实效，以钉钉子精神做实做细做好每一项工作，落实好每一件事情，完成好每一项任务，用实干推进湛江全面振兴发展！

　　各位代表，历经改革开放40年的洗礼，湛江大地生机勃发、活力四射。走进新时代，机遇垂青我们；肩负新使命，发展考验我们；踏上新征程，人民期待我们！让我们紧密团结在以习近平同志为核心的党中央周围，在习近平新时代中国特色社会主义思想指引下，坚决把思想和行动统一到党的十九大精神上来，高举中国特色社会主义伟大旗帜，在市委坚强领导下，提高发展自觉，增强发展自信，奋力拼搏，后发赶超，为决胜全面建成小康社会、加快建设北部湾中心城市和省域副中心城市，打造广东新的增长极不懈奋斗！
